# Bounding the Multi-Scale Domain in Numerical Modelling and Meta-Heuristics Optimization: Application to Poroelastic Media with Damageable Cracks

**DOI:** 10.3390/ma14143974

**Published:** 2021-07-16

**Authors:** Albert Argilaga, Efthymios Papachristos

**Affiliations:** 1 MOE Key Laboratory of Soft Soils and Geoenvironmental Engineering, Zhejiang University, Hangzhou 310058, China; 2 Ecole Centrale de Nantes, Institut de Recherche en Génie Civil (GeM), UMR6183, 44321 Nantes, France; efthymios.papachristos@gmail.com

**Keywords:** meta-heuristics, asymptotic homogenization, periodic micro-structure, Particle Swarm Optimization, multi-scale, FEM, in-simulatio, constitutive law

## Abstract

It is very common for natural or synthetic materials to be characterized by a periodic or quasi-periodic micro-structure. This micro-structure, under the different loading conditions may play an important role on the apparent, macroscopic behaviour of the material. Although, fine, detailed information can be implemented at the micro-structure level, it still remains a challenging task to obtain experimental metrics at this scale. In this work, a constitutive law obtained by the asymptotic homogenization of a cracked, damageable, poroelastic medium is first evaluated for multi-scale use. For a given range of micro-scale parameters, due to the complex mechanical behaviour at micro-scale, such multi-scale approaches are needed to describe the (macro) material’s behaviour. To overcome possible limitations regarding input data, meta-heuristics are used to calibrate the micro-scale parameters targeted on a synthetic failure envelope. Results show the validity of the approach to model micro-fractured materials such as coal or crystalline rocks.

## 1. Introduction

Natural or composite materials, are often characterized by micro-structure [1,2,3]. At a range of different loading conditions, the constitutive response of the micro-structure might affect or even govern the bulk constitutive response of the material in the macro-scale, while in other conditions it might not play a significant role. However, modeling the material at very small scales is computationally costly (or prohibiting). At cases where the micro-structure is periodic (or quasi-periodic) the information of the micro-scale response can be passed to the macroscopic description of the material through a homogenization approach [4,5,6,7,8,9], and solved on a generic micro-structural cell e.g., [10]. The homogenized constitutive laws, also called double-scale laws, allow for a modelling of such materials at the macro-scale which is more computationally efficient. Indicative examples of the need for double-scale models is for the description of masonry structures [11,12], coalbed reservoirs [13,14], shales [15,16] as well as other rocks [17]. These materials are characterized by a porous matrix (material skeleton) stratified by damageable cracks.

**Figure 1:**
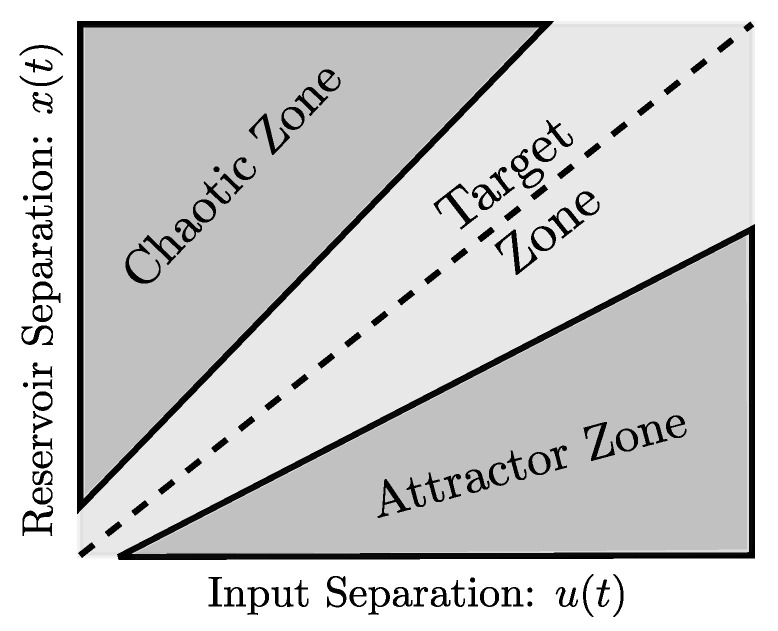
Separation Ratio Graph based on [34]. Graphical representation of the phase transition between chaos and order. Systems in the target zone are said to possess both a good separation property and ideal dynamic behaviour to produce optimal reservoirs.

Physical systems (e.g., materials) can perform simulations with a degree of complexity unattainable by conventional von Neumann computers [18]. Nonetheless, due to the number of variables and uncertainty present in physical systems, control and reproducibility and even measurement of such experiments are difficult tasks. With the increasingly available computing power of classical computers, accurate discretizations of Partial Differential Equation (PDE) problems can serve the needs of researchers: a new experimental domain is born: *in-simulatio* experimentation [19]. In recent years artificial intelligence (AI) techniques have become very popular to solve problems which require a high level of cognition, e.g., image recognition, audio signal discrimination, autonomous driving, natural hazard mitigation [20], materials constitutive modelling ([21,22,23,24,25,26,27], among others), but also to solve physical problems which where traditionally the realm of PDEs (see [28,29,30,31] for example). AI paradigms are implemented in classical von Neumann computers because of their degree of developement compared to physical computing systems but those architectures may not be the optimal solution for such applications. The interest in developing computing systems combining physical and classical computers has been growing in parallel to the development of AI; physical computers are meant to provide high level, fast, low energy demanding and non deterministic computations while the classical computer layers can treat the data in different stages of the computation like pre or post processing. While any physical system can be used to perform computations, some characteristics are desirable in order to maximize computing power, this relates to the quantity of chaos and order in the system [32]. A perfectly ordered system will be attractor based and will not provide any result which we do not already know from the inputs, while an excessively chaotic system will output a result that cannot be interpreted. The order-chaos range in which powerful computations are possible appears to be very narrow (Figure 1), and it seems that some biological systems, e.g., distribution of neuronal avalanche sizes [33], belong to this range.

Multi-scale modelling is an increasingly popular trend taking advantage of classical computers [35,36,37,38,39,40]. This paradigm uses *in-simulatio* numerical results in order to feed a higher hierarchical model instead of using classical mathematical descriptions. This has the advantage of producing more feature rich results without the hassle of dealing with experiments in a hybrid numerical-experimental setup. Indeed, in a multi-scale approach, the micro-scale computation is the *in-simulatio* analogous of an *in-materio* computer. An asymptotic homogenization and the related double-scale model for poroelastic media with damageable cracks were previously developed [41]. Compared to numerical homogenization approaches such as direct micro–macro-techniques [39,42], the asymptotic homogenization theory permits equivalent properties to be obtained and allows an analytic and a numerical approach to be combined. The homogenized problem can be solved on a generic micro-structural cell (solved using, e.g., finite elements) so that the homogenized macroscopic properties are finally obtained. The obtained homogenized macroscopic properties remain valid until a change in the micro-structural cell requires an update of the homogenized solution, e.g., because of crack damage. Another alternative could be the use of Machine Learning (ML) methods for either black-box or physics-informed homogenization. ML methods usually offer much lower computational cost regarding the estimation of the homogenized operator, but as a trade off they may lack in rationality (black-box) and they need a relatively large amount of input data (micro-structure simulations in this case) for non-overfitting training.

For further studying the applicability of the model presented in [41], the different response regimes of the physical system (the material) must be identified and bounded. Following this reasoning, the characteristics of micro-scale numerical models can be compared to those of *in-materio* computing kernels, i.e., if the micro-scale behaviour is too simple (predictable), it will not present an advantage with respect to a classical phenomenological expression yet if too complex (chaotic) its output will not provide extra useful information. In the following, we are first exploring the validity bounds of the model and identifying the different regimes into fractures-dominated, inter-play matrix-fractures, matrix-dominated. Then the failure envelopes are obtained and the continuity and differentiability of the model is studied. Finally, the micro-scale needs to be calibrated. In practice, accessing direct measurements of a micro-scale’s mechanical properties at the nanometer scale is not possible. In general, obtaining the 3D micro-scale configuration of materials is possible by techniques such as X-ray tomography [43], water distribution can be determined with neutron tomography [44], bulk force networks can be deduced using photoelasticity [45], and it is even possible to obtain metrics of the contacts’ fabric [46,47]. Nevertheless, it is still challenging to obtain direct measurements of force at the contacts’ level. Thus, the main geometric characteristics of the micro-scale can be known, but in most cases the precise calibration of its components needs to be obtained from the macro-scale material response.

The characteristics that render the micro-scale useful for multi-scale frameworks also make it untreatable with classical gradient methods like Newton method [48]. In recent years, meta-heuristics based on the mimicking of natural systems and cooperative populations have shown different degrees of success in particularly challenging optimization problems [49,50,51]. Particle Swarm Optimization (PSO) is proposed in the present work as a meta-heuristics approach [52] to find minima of the objective function resulting from the micro-scale homogenization fitting with a target response.

The purpose of this work is to provide a micro-scale model that can be directly used in a multi-scale numerical approach, establish its validity range based on in-materio computing and provide a calibration method able to overcome the challenges resulting from the particularities of the micro-scale. The article is organized as follows: Section 2 presents the constitutive equations of the micro model and its numerical implementation, Section 3 showcases different failure envelopes and proposes a well suited method to characterize the micro-scale in view of a multi-scale application, Section 4 exposes the meta-heuristics optimization results and Monte-Carlo analysis, the paper ends with discussion Section 5 and conclusions in Section 6.

## 2. Description of the Micro-Scale Problem

In the following, we consider a 2-D micro-scale structure that consists of a porous elastic matrix and a crack network. The crack opening presents a linear elastic response with opening and undergo a nonlinear damage evolution. The cell is considered to be *x* and *y* periodic. This configuration can be representative of many materials including coal, crystalline rocks, composites and more.

### 2.1. Constitutive Equations of the Homogenized Problem

The constitutive equations of the studied micro-scale were obtained using asymptotic homogenization technique [53] in the framework of small strains using asymptotic expansions [54], full description of the expansions can be found in [41]. The original problem is hydromechanical, however, the hydraulic and mechanical parts can be totally uncoupled and only the mechanical part is considered in the present paper.

The strong form of the mechanical problem after homogenization reads:

(1) $${\mathrm{div}}^{y}\sigma^{\left(0\right)}=0\;,\;\mathrm{in}Y$$

(2) $$\sigma^{\left(0\right)}\cdot \vec{n}={\vec{T}}^{\left(0\right)}\;,\;\mathrm{on}\Gamma^{Y}$$

(3) $$\sigma^{\left(0\right)}=c:\left(\epsilon^{x}\left({\vec{u}}^{\left(0\right)}\right)+\epsilon^{y}\left({\vec{u}}^{\left(1\right)}\right)\right)-p^{\left(0\right)}\alpha$$

(4) $${\vec{T}}^{\left(0\right)}=G\cdot \left[\left[{\vec{u}}^{\left(1\right)}\right]\right]-p^{\left(0\right)}\vec{A}\;,\;\mathrm{on}\Gamma^{Y}$$

(5) $${\vec{u}}^{\left(1\right)},\sigma^{\left(0\right)}\;Y-\mathrm{periodic}$$

where $\vec{u}$ is the displacement field, macro ${\vec{u}}^{\left(0\right)}$ and micro ${\vec{u}}^{\left(1\right)}$, σ is the total Cauchy stress tensor and $p^{\left(0\right)}$ is the macro pore pressure. *c* is the fourth order tensor of elastic stiffness and α is the second order tensor of Biot coefficients. Equation (4) is similar to Equation (3) in this case for the crack network, *G* and $\vec{A}$ being the elastic stiffness of the cracks and its tensor of Biot coefficients. Since both matrix and crack Biot coefficients are solely affected by macro pore pressure $p^{\left(0\right)}$ their contributions can be obtained by linear combination of ${\vec{u}}^{\left(0\right)}$; for sake of simplicity Biot coefficients will be taken as zero in the present paper.

The cracks separating the porous parts of the medium are very soft. That means that the lips of the cracks can slide and open and, in order to maintain coherence, the stress vector $\vec{T}=\sigma \cdot \vec{n}$ is continuous on the cracks. The displacement field $\vec{u}$ is then discontinuous on the cracks and its jump ${\vec{u}}^{+}-{\vec{u}}^{-}$ through a crack where ${\vec{u}}^{+}$ is the value of $\vec{u}$ on the side toward which $\vec{n}$ points and ${\vec{u}}^{-}$ is the value of $\vec{u}$ on the opposite side, is denoted by $\left[[\vec{u}]\right]$.

#### Damage

Damage is introduced in the micro-structure in order to model the degradation and reduction of mechanical properties observed in real materials. Here, it is considered that the damage is concentrated solely in the crack network. In this case, Equation (4) becomes (through the corresponding expansions):

(6) $${\vec{T}}^{\left(e\right)}=\left(1-d^{\left(0\right)}\left(\tau \right)\right)G\cdot \left[\left[{\vec{u}}^{\left(1\right)}\left(\tau \right)\right]\right]-p^{\left(0\right)}\left(\tau \right)\vec{A}$$

with

(7) $$d^{\left(0\right)}\left(\tau \right)=\underset{0\leq \rho \leq \tau }{\sup}f\left(\frac{∥\;\left[\left[{\vec{u}}^{\left(1\right)}\left(\rho \right)\right]\right]\;∥}{\Delta_{n}^{\left(e\right)}}\right)$$

where *f* is the damage function [55]:

(8) $$z\overset{f}{\to }f\left(z\right)=\left\{\begin{array}{cc} z\left(2-z\right) & 0\leq z<1\\ 1 & 1\leq z \end{array}\right.$$

where τ is the time-history variable of the damage parameter $d^{\left(0\right)}$, ρ is the time-history variable of the displacement field ${\vec{u}}^{\left(1\right)}\;$ in the crack network, $\Delta_{n}^{\left(e\right)}$ is a length-like feature of the material of the cracks. At initial time t=0, the porous medium is assumed to be unloaded, unstressed, unstrained and undamaged which means that the initial value of the damage parameter is 0.

### 2.2. Numerical Implementation

**Figure 2:**
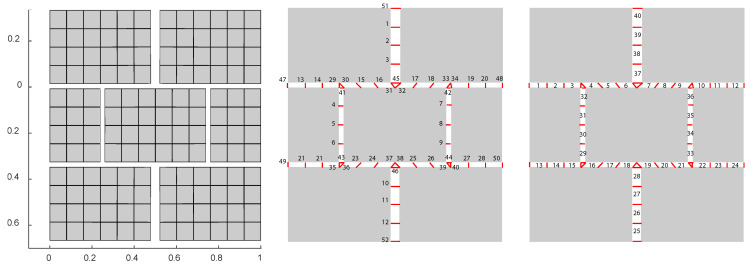
Geometry of the micro-scale configuration Finite Element Mesh, matrix elements with unit cell adimensional axes (**left**), crack network links (**center**) and crack network elements (**right**). Cracks are totally closed in the initial configuration, figure shows cracks with opening for convenience of representation.

The weak formulation of the previous equations is implemented in a 2D Finite Element Model (FEM) developed using Matlab [41]. A base cell geometry is chosen consisting of 144 2D 4-node quadrangular elements modelling the matrix grains (Figure 2, left), 52 crack jump links (Figure 2, center), and 40 linear 2-node elements modelling the cracks (Figure 2, right), 215 nodes in total.

The elastic properties of the matrix can be defined by the Lame constants: λ=1.442GPa and μ=0.961GPa, i.e., Young’s modulus E=2.5GPa and Poisson’s ratio ν=0.3. This constants have been chosen according to and for validation purposes [56]. The problem being non-linear and path-dependent, it is loaded step by step and an iterative Secant method approach is used at each step to find a solution. In order to obtain the failure envelopes, a loop parallelization *parfor* in Matlab has been used to accelerate the computation of concurrent loading paths. In the next section, and before obtaining the failure envelopes, the homogenized stiffness coefficients have been studied to establish the initial bounds of the input variables.

### 2.3. Initial Constriction of Crack Stiffness

**Figure 3:**
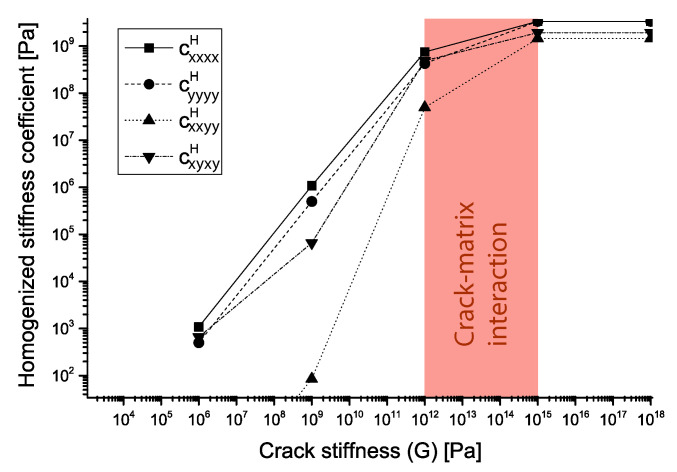
Homogenized stiffness function of crack stiffness. Below the red area crack stiffness is dominant and above porous matrix is dominant. The red area corresponds to a material with both crack and porous matrix contribution to the response.

The study range is initially constrained to values of the crack stiffness between $G=10^{12}$ Pa and $G=10^{15}$ Pa, in which, the homogenized coefficients present contribution from both crack and matrix properties (Figure 3). This is done in the spirit of *in-materio* computing: the range $G=[10^{12}$∼$10^{15}]$ Pa maximizes dissimilarity between output states and a linear combination of the inputs.

**Figure 4:**
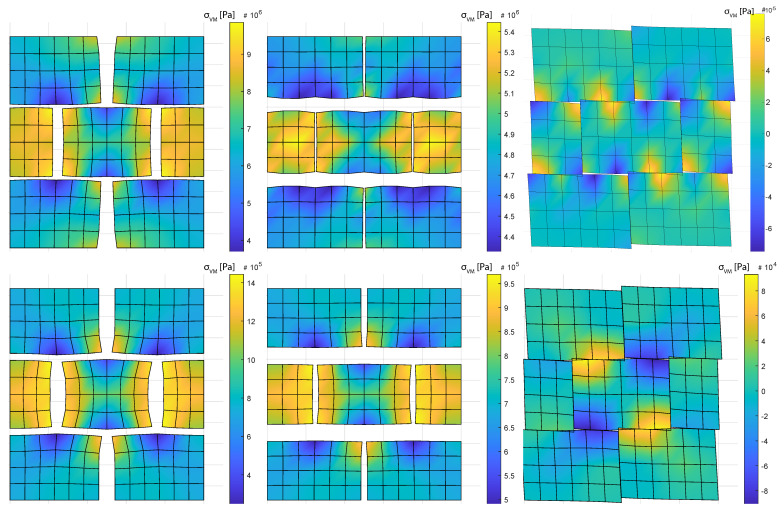
Configurations for a macro deformation ϵ=0.005 in all DoF, including crack pore pressure. Colormap represents von Misses strain in Pascals. Crack stiffness: $G=10^{13}$ GPa (**top**) and $G=10^{14}$ GPa (**bottom**). Deformation magnification: 50×.

The range *G* = [$10^{12}$∼$10^{15}$] Pa is valid for the previously defined elastic properties of the matrix μ and λ. Outside this range the problem becomes either crack-driven ($G<10^{12}$ Pa) or matrix-driven ($G>10^{15}$ Pa). Micro-scale deformation configurations are shown for two values of crack stiffnesses and no damage: $G=10^{13}$ Pa (Figure 4, top) and $G=10^{14}$ Pa (Figure 4, bottom) for an arbitrary magnitude of strain in each of the degrees of freedom of the macro-scale deformation space. This initial crack stiffness constriction is used to bound the failure envelope study in the next section.

## 3. Failure Envelopes

This section presents a series of failure criteria in order to determine the best suited one for the present application. In a bifurcation or strain localization problem, Rice criterion would be the natural choice to determine onset of localization [57,58]. Neither well-posedness of the problem nor localization are of concern here since regularization techniques can be applied on the top of a multi-scale scheme [59,60,61,62]. The purpose of the present work is to provide a method to calibrate a constitutive law aiming at hierarchical multi-scale modelling rather than describing bifurcation or onset of localization.

### 3.1. Maximum Stress Failure Criterion

The failure criterion is based on the attainable maximum stress in the coordinate reference system (x,y). If any of the three components of the stress tensor $\sigma_{xx}$, $\sigma_{yy}$ or $\sigma_{xy}$ absolute value diminishes in a time step (Equations (9)–(11)) the configuration is considered to be failed.

(9) $$∥\sigma_{11}∥<\underset{0\leq \rho \leq \tau }{\sup}∥\sigma_{11}\left(\tau \right)∥$$

(10) $$∥\sigma_{22}∥<\underset{0\leq \rho \leq \tau }{\sup}∥\sigma_{22}\left(\tau \right)∥$$

(11) $$∥\sigma_{12}∥<\underset{0\leq \rho \leq \tau }{\sup}∥\sigma_{12}\left(\tau \right)∥$$

**Figure 5:**
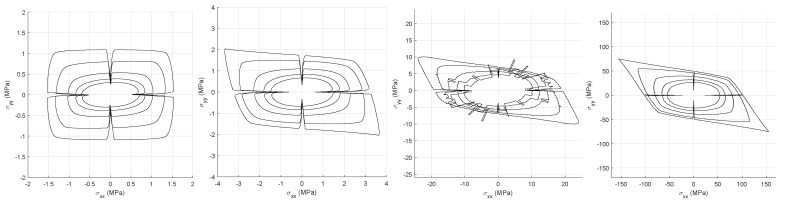
Failure envelopes for different crack stiffnesses, maximum stress failure criterion. Respectively from **left** to **right** $G=10^{12}$ Pa–$10^{13}$ Pa–$10^{14}$ Pa–$10^{15}$ Pa. The concentric envelopes correspond to different applied macro shear. The macro shear spans from zero, for the outmost envelope, until 0.200 for the innermost, with an increment of 0.040.

It is assumed that the loading is at constant rate and euclidean. Failure envelopes are generated for a series of crack stiffnesses *G* (Figure 5). The issue with this criterion is the creation of artefacts due to close to zero stress components when loading parallel to the coordinate system. Two features can already be observed: (a) higher homogenized stiffnesses and orthotropic response for higher *G* (crack driven biased response) and (b) crack stiffness $G=10^{14}$ Pa gives a characteristic non-continuous stress envelope more pronounced for the cases with higher $\sigma_{xy}$.

### 3.2. Von Misses Failure Criterion

To avoid previous artefacts von Misses distortion energy (Equation (12)) is used as failure criterion in the following envelopes. The material is considered to fail when the distortion energy diminishes between two consecutive loading steps (Equation (13)).

(12) $$vm_{c}=\sigma_{11}^{2}+\sigma_{22}^{2}-\sigma_{11}\sigma_{22}+3\sigma_{12}^{2}$$

(13) $$vm_{c}<\underset{0\leq \rho \leq \tau }{\sup}\left(vm_{c}\left(\tau \right)\right)$$

**Figure 6:**
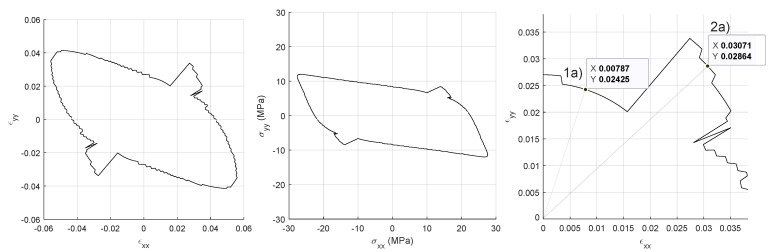
Failure envelopes with crack stifness $G=10^{14}$ Pa. von Misses energy criterion: strains (**left**), stresses (**center**). Coordinates in the strain plane to extract configurations of failure region 1a and 2a. (**right**).

From the previous cases, only the case for crack stiffness $G=10^{14}$ Pa is considered for further study. In addition, macrostrain $\epsilon_{xy}$ is kept equal to zero for sake of shortness. The envelope presents a fish-like silhouette with a discontinuity both in the stress and strain envelopes around the path $\epsilon_{xx}=\epsilon_{yy}$ (Figure 6, left and center).

**Figure 7:**
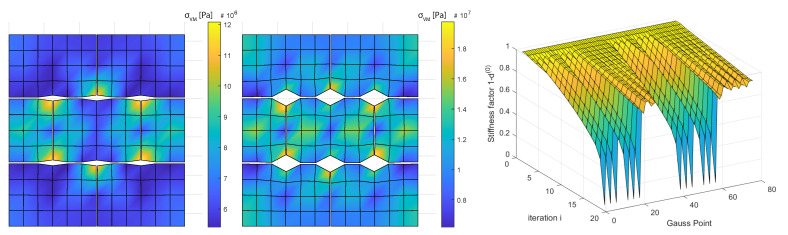
Failure according to von Misses energy criterion. From **left** to **right**: region 1a before failure, one step after failure and stress reduction after damage law expansion. Micro-scale deformation magnification 10×. $G=10^{14}$ Pa.

Outside this region the envelope presents saw like profile, this can be put down to the resolution of the time stepping in the $\epsilon_{xx}-\epsilon_{yy}$ space, rather than the switching between different failure mechanisms. Similar results can be observed for crack stiffnesses in the range *G* = [$1\times 10^{13}$∼$1\times 10^{14}$] Pa (results not presented). One drawback of using von Misses failure criterion is that it assumes that yield happens due to accumulated distortion energy, meaning that it is insensitive to isotropic compression. This is obviously not the case for the present model in which isotropic compression does cause crack damage. Two points in the $\epsilon_{x}-\epsilon_{y}$ space are chosen corresponding to the different parts of the failure envelope (Figure 6, right). In the following, the lower strain failure space will be denominated *Failure Region 1a* and the higher one *Failure Region 2a*, simulations are run for both points. The loading is scaled accordingly in each simulation so the time steps have the same size as the ones used to obtain the envelopes. Results show the configuration one loading step before and one immediately after failure according to von Mises distortion energy criterion. Failure Region 1a presents slight opening of the horizontal longitudinal cracks in the six intersections with the vertical cracks before the failure (Figure 7, left), after the failure those crack links snap open (Figure 7, center).

**Figure 8:**
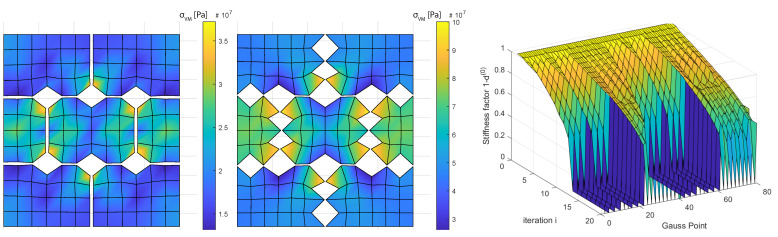
Failure according to von Misses energy criterion. From **left** to **right**: region 2a before failure, one step after failure and stress reduction after damage law expansion. Micro-scale deformation magnification 10×. $G=10^{14}$ Pa.

Failure Region 1a has a ratio $\epsilon_{yy}/\epsilon_{xx}=3.1$, which makes it natural to be the horizontal cracks the first ones to open and fail. It is also remarkable that the horizontal cracks do not open longitudinally but only in the intersections with other cracks. The stiffness factor $1-d^{\left(0\right)}$ plots present the stiffness reduction for the Gauss Points of the discontinuity network (80 Gauss Points) (Figure 7, right), this corresponds to 52 crack node links minus 12 links sharing the same nodes: 40 crack elements (Figure 2, right) and 2 Gauss Point per crack element. Given Gauss Points are affected by stiffness reduction more rapidly than others during the 20 time step lading until 12 of them totally fail and this triggers the von Misses criterion. These 12 points can be identified in (Figure 2, center). Same outputs in the Failure Region 2a present an opening of the horizontal cracks in the intersection points similar to the previous case (Figure 8, left).

Nevertheless, due to the different ratio $\epsilon_{yy}/\epsilon_{xx}=0.93$, this crack damage does not cause failure according to the von Misses criterion and the loading can continue until vertical cracks open along with additional points in the horizontal cracks (Figure 8, center) that did not open in the Failure Region 1a case. Similarly to the Failure Region 1a case, cracks did not open continuously but only in selected points with a clear mesh dependency pattern. In (Figure 8, right), the Gauss-points seem to undergo a similar process until iteration 12 (60% final loading), when the same 12 Gauss Points as in Failure Region 1a totally fail but due to the different $\epsilon_{yy}/\epsilon_{xx}$ ratio the von Misses criterion is not triggered, the loading can continue until vertical crack links fail together with some additional horizontal crack links.

**Figure 9:**
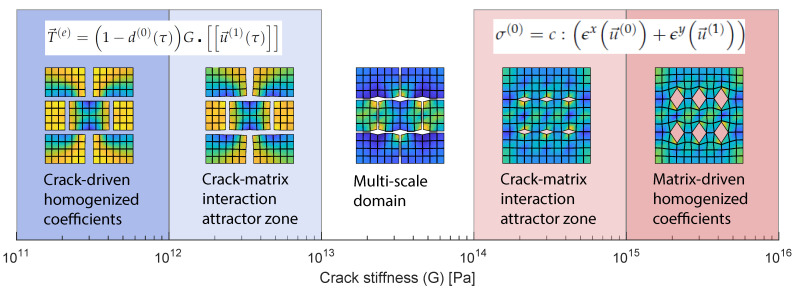
Micro-scale configuration crack stiffness range using von Misses failure criterion.

After obtaining the different failure envelopes for different crack stiffnesses *G* the range presented in (Figure 3), which was issued from previous results, can be further refined (Figure 9).

According to the von Misses distortion energy criterion, the range of *G* where the crack-matrix coupling exhibits interesting features for multi-scale modelling (switching failure modes) is *G* = [$1.0\times 10^{13}$∼$1.0\times 10^{14}$] Pa. The upper range of *G* will be further clipped down to $G=2.70\times 10^{13}$ Pa to avoid computationally expensive simulations of unbreakable micro-scales: for stiff crack networks and specific loading paths the resulting damage pattern turns the micro-scale into a matrix based spring like geometry with much softer homogenized properties than the matrix itself, this kind of configuration is able to undergo large strain inputs without further loading of the crack network. See animations of some unbreakable micro-scale mechanisms in the supplemented data.

### 3.3. Stress Ratio to Peak Failure Criterion

**Figure 10:**
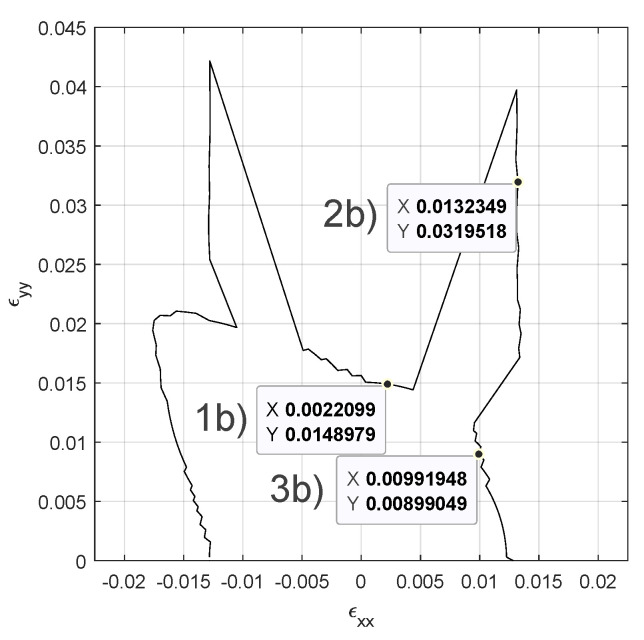
Failure envelope with crack stiffness $G=2.00\times 10^{13}$ Pa. Stress ratio to peak failure criterion. Coordinates to extract configurations of failure regions 1b–2b–3b.

Due to the shortcomings of the previous failure criterion regarding isotropic loading damage and unbreakable micro-structures, yet a new one is proposed based on the decrease of force modulus (Equation (14)) after peak: during a loading history, when the resulting force magnitude decreases from a given peak ratio the sample is considered to be failed (Equation (15)):

(14) $$fm_{c}=∥\sigma_{ij}∥$$

(15) $$fm_{c}<r_{fm}\left(\underset{0\leq \rho \leq \tau }{\sup}\left(fm_{c}\left(\tau \right)\right)\right)$$

where $r_{fm}$ is the peak force ratio. By taking the force magnitude instead of the cartesian force components, previous artefacts in the failure envelope are avoided. Since the output of this model is meant to be injected into a multi-scale framework, a failure criterion as maximum force or alike does not necessarily mean loss of controllability [63]. When the maximum stress criterion is fulfilled in a material point of a boundary value problem the stress redistributes in its neighbourhood meaning that this point does not necessarily collapse under constant stress conditions or cause the loss of controllability of the macro-scale problem. Therefore the introduction of the ratio $r_{fm}$ in Equation (15), this allows to obtain a failure envelope more representative of multi-scale coupling. The value $r_{fm}=0.8$ is used in the following, this value has been chosen as a compromise between high values close to $r_{fm}=1$ which fail immediately after the peak and low values close to $r_{fm}=0$ which create unbreakable micro-scales. A failure envelope is presented for the micro-scale parameters: $\Delta_{n}=0.0050$, $\mu =961\times 10^{6}$, $\lambda =1442\times 10^{6}$, $G=2.00\times 10^{13}$ and three failure regions are marked for later analysis (Figure 10).

**Figure 11:**
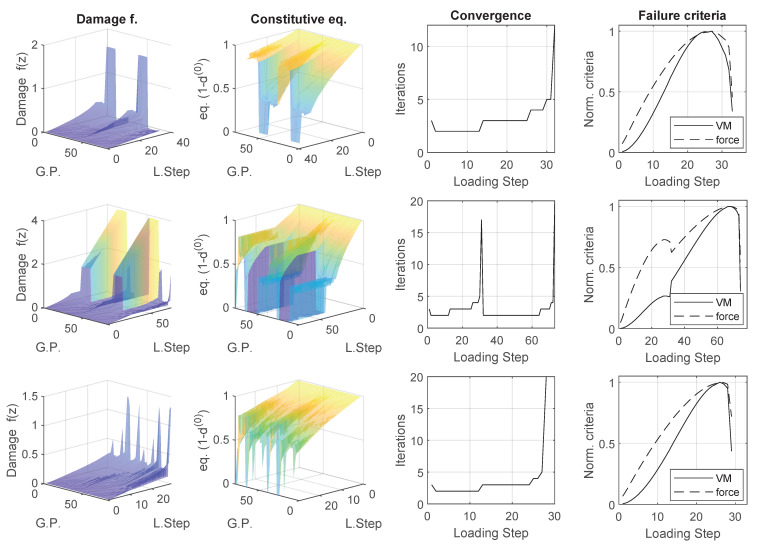
Stress ratio to peak failure criterion results. From top to bottom failure region 1b, 2b and 3b. From left to right: damage parameter f(z) (Equation (8)), constitutive equation affecting Gauss Points $\left(1-d^{\left(0\right)}\right)$ (Equation (7)), iterations for convergence of the nonlinear problem at each loading step and normalized failure criteria (von Misses and Stress ratio).

Results of loading paths for the three failure region points are presented in (Figure 11). From top to bottom they are: failure region 1b, 2b and 3b. Figures from left to right show the damage parameter f(z) (Equation (8)), constitutive equation affecting Gauss Points after damage $\left(1-d^{\left(0\right)}\right)$ (Equation (7)), iterations for convergence of the nonlinear problem at each loading step and normalized failure criteria: von Misses distortion energy (VM) and Stress ratio to peak failure criterion.

Failure regions 1b and 3b show a similar behaviour: damage function in the Gauss Point network progressively increases with a slight acceleration over the loading until suddenly one of the horizontal cracks opens in a strain-localization-like phenomena. Number of iterations for convergence gives an idea of the possibility of bifurcation, their value is 5 or below until the moment of failure indicating a brittle failure of the micro-structure, this can be also observed in the steep descent of both norms of failure criteria. Failure region 2b presents a different history. Damage function evolves to a strain localization in a horizontal crack around iteration 30, same as in the previous cases, but this is not enough to trigger the failure criterion, loading continues until a part of the vertical crack network fails. Region 2b behaviour can also be identified in the number of iterations for convergence as well as in the norm of von Misses and Stress ratio to peak failure criterion.

With a failure envelope representative of the present micro-structure application, the model is ready to be calibrated. Next section proposes a calibration method and presents a calibration using synthetic data.

## 4. Particle Swarm Optimization

Due to the very same characteristics that make the present *in-simulatio* micro-scale appropriate for multi-scale, the law cannot be easily tackled using traditional gradient techniques. Particle Swarm Optimization (PSO) is proposed as an alternative optimization approach to calibrate the micro-scale. An alternative could be to launch a large campaign of micro-scale simulations to obtain adequate data for the training of a ML algorithm and replace the asymptotic homogenization with it.

PSO is a meta-heuristic used to find optima of highly nonlinear, discontinuous, non differentiable or functions containing random variables. In contrast to gradient-based optimization methods it does not require the differentiation of the objective function and is less prone to get stuck in local minima. A campaign of optimization runs is set up in order to find an appropriate swarm size. To avoid high computational costs the swarm size is set to a very small value: SwarmSize=8 and progressively increased until getting satisfactory results, this search is done with a low resolution of the objective function: the damage surface is discretized into 8 points. Once the results of the optimization are successful the swarm size is kept constant and the resolution of the objective function progressively increased in order to verify convergences and avoidance of local minima in a refined non differentiable target function. All optimizations are restricted to 100 iterations with additional stopping criteria in case of no convergence. Table 1 presents a summary of the used PSO parameters:

### 4.1. Objective Function

**Figure 12:**
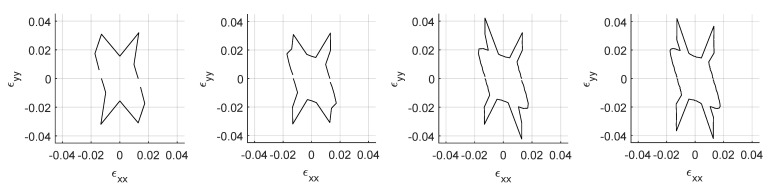
Failure surfaces for different function resolutions. From left to right: 8–16–32–64 points.

Four failure envelopes using the stress ratio to peak failure criterion are obtained with different degrees of $\epsilon_{11}$–$\epsilon_{22}$ resolutions (Figure 12).

These are utilized as target functions to evaluate the goodness of fit of the optimizations. Objective functions correspond to the Top (or Macro-) level emerging constitutive response and the Bottom (or micro-) level is optimized based on them. The used resolutions are 8, 16, 32 and 64 points for half of the $\epsilon_{11}$–$\epsilon_{22}$ space. Due to the nature of the constitutive law the other half is symmetric thus an evaluation of the micro-scale response for those loading paths is not needed. The damage envelope with only 8 points is very abstract and does not provide a very good representation of the actual surface, this minimal value has been chosen to allow faster runs of the optimization scheme before applying it to higher resolutions. It is also representative of a calibration with limited experimental data availability. A resolution of 32 points already displays most of the features of the damage envelope with the 64 points refining even more some details. Next section presents the results of optimizations with different swarm sizes and objective function resolutions.

### 4.2. Increasing Swarm Size Results

**Figure A1:**
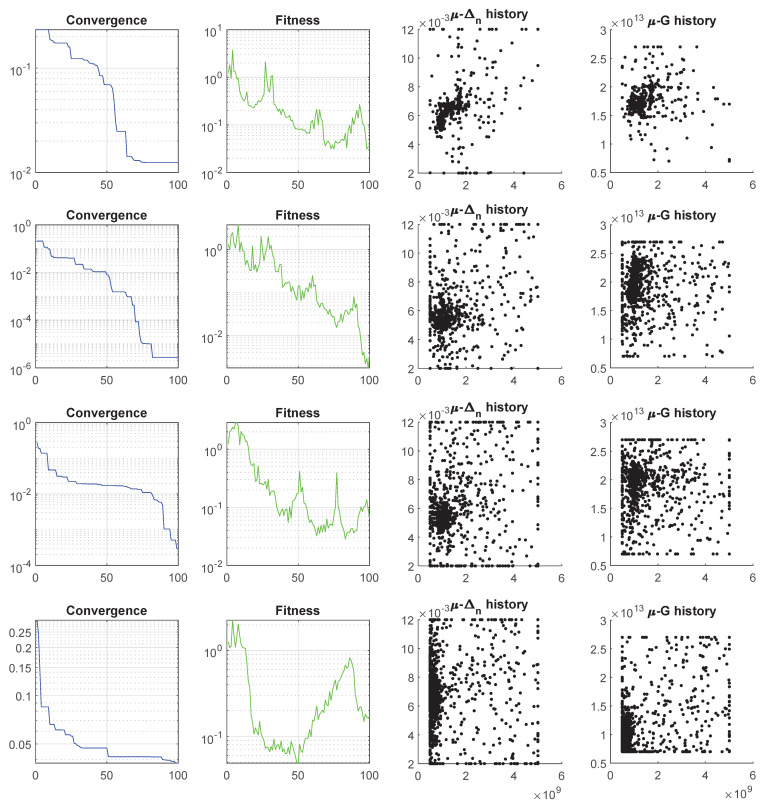
Blue plot is the convergence of the PSO algorithm (minimum value of f(x)), green plot is the fitness of the population (mean value of f(x)) both plots horizontal axes are the PSO iterations. The two scatter plots represent different planes in the optimization variable space, $\mu -\Delta_{n}$ and μ−G. The axes are in the units of the corresponding variable, Pa or adimentional for $\Delta_{n}$. Swarm Population from top to bottom: 8–16–24–32. Objective function f(x) resolution: 8 points.

A first optimization with SwarmSize=8 and f(x) Resolution=8 presents problems with a local minima trap (Figure A1, 1st row) and does not converge after iteration 65, fitness is also poor. Scatter plots (Figure A1, 1st row) present the superposition of the swarm positions along the optimization, the plots do not provide information about the swarm evolution during the optimization but rather the areas where the search has been more intense. Log range of the different optimization variables *x* (Figure A3, 1st row) show some convergence but not to the correct value (in particular *G*). Swarm size is increased to 16 (Figure A1, 2nd row), Swarm Sizes are picked to be multiples of 8 to maximize the efficiency of the PSO parallelization since it is running on 8 CPU-threads. Convergence is much better than the previous case, achieving a minimum value of the objective function $f\left(x\right)=2.608\times 10^{-6}$ already at iteration 83, convergence does not progress from iteration 83 to 100 but the fitness of the population does due to the speed updates with the new optimal value. Scatter plots of the *x* vector positions show an intense search for optimal *G* along a constant μ. Log range of the different optimization variables *x* (Figure A3, 2nd row) show convergence and this is accelerated after finding the minimum around iteration 83. Swarm size is increased to 24 (Figure A1, 3rd row). Convergence does not reach as low residual values as before and it presents a plateau of slow convergence between iterations 30 and 80 which looks like a *cross of the desert* by the particles during which no local minima where found. Similarly to the previous case, scatter plots of *x* show an intense search for optimal *G* along a constant μ. Log range of the different optimization variables *x* (Figure A3, 3rd row) show convergence with a slight divergence of *G* the last 10 iterations, probably due to a late global swarm convergence not reaching very low values. Swarm size is increased to 32 (Figure A1, 4th row). Convergence is very poor and fitness increases after iteration 50. Scatter plots of *x* show that the swarm particles are stuck in the boundaries of the *x* domain. Log ranges show high values with oscillations and no convergence (Figure A3, 4th row).

### 4.3. Increasing Objective Function Resolution Results

**Figure A2:**
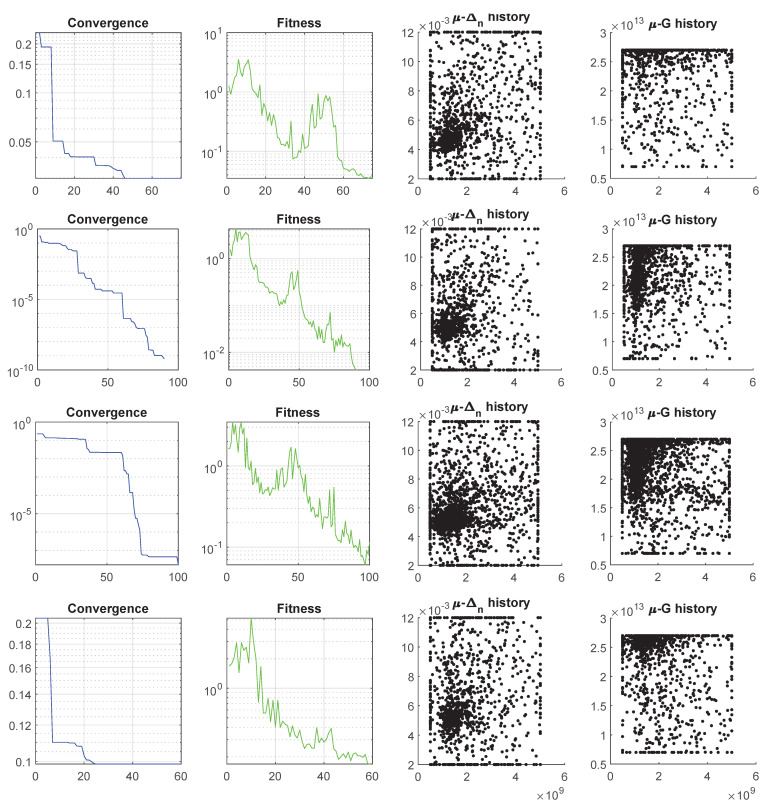
Blue plot is the convergence of the PSO algorithm (minimum value of f(x)), green plot is the fitness of the population (mean value of f(x)), both plots horizontal axes are the PSO iterations. The two scatter plots represent different planes in the optimization variable space, $\mu -\Delta_{n}$ and μ−G. The axes are in the units of the corresponding variable, Pa or adimentional for $\Delta_{n}$. Swarm Population: 40. Objective function f(x) resolution from top to bottom: 8–16–32–64 points.

Swarm size is increased to 40 particles (Figure A2, 1st row). Convergence is not good and the progress stalls after iteration 46 due to falling into a local minimum trap, fitness is also poor and the scatter plots show how the variable *G* search efforts are concentrated in the upper boundary near $G=2.7\times 10^{13}$ Pa. Given the slow convergence after iteration 10, it seems that around that time the swarm was induced towards the wrong local minimum. An optimization with larger swarm population presenting worse result than before can seem contradictory, but it must be reminded that meta-heuristics like PSO use stochastic functions to define initial positions and velocities, thus results are not determinist. Log range of the different optimization variables *x* (Figure A4, 1st row) show convergence, not as good as the previous case for $\Delta_{n}$ and μ, but in the same order of magnitude for *G* which agrees with the data shown in the scatter plot (Figure A2, 1st row μ−G). The results for Swarm size 40 particles are presented in (Figure A2, 2nd row). Resolution of objective function f(x) is increased to 16. Convergence decreases without major stall periods along the 100 iterations to a very low value of the residual of $6.136\times 10^{-10}$. Nevertheless the value of *x* is not as good as the one obtained in the simulation with swarm size 24 (simulation #3), seemingly due to the existence of a local minimum very close to the global one. Log ranges of *x* (Figure A4, 2nd row) present good convergences. Swarm size 40 particles (Figure A2, 3rd row). Resolution of objective function f(x) is increased to 32. Convergence is slow until iteration 60 and then in quickly converges to a residual of $1.635\times 10^{-10}$. Similarly to the previous case and probably because the same reason the value of *x* is not as good as the one obtained in simulation #3. Log ranges of *x* (Figure A4, 3rd row) present good convergences with some strong oscillations in *G*. Swarm size 40 particles (Figure A2, 4th row). Resolution of objective function f(x) is increased to 64. Convergence is slow after iteration 8 and and it stalls at iteration 25 with a residual of 0.0986. Scatter plots show that this is due to the swarm getting stuck in the boundary $G=2.7\times 10^{13}$ Pa. Log ranges of *x* (Figure A4, 4th row) show no convergence.

### 4.4. Summary Particle Swarm Optimization Results

**Figure A3:**
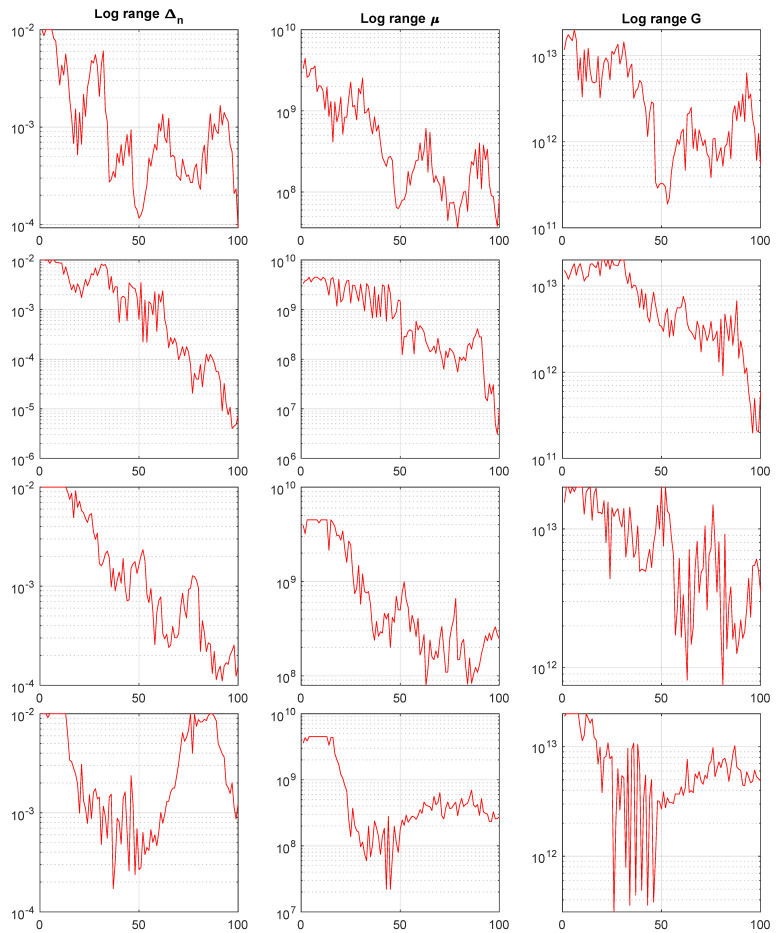
The three columns represent the evolution of each optimization variable ranges, respectively: $\Delta_{n}$, μ and *G*. The horizontal axes are the PSO iterations and the vertical axes are in the units of the corresponding variable, Pa or adimensional for $\delta_{n}$. Swarm Population from top to bottom: 8–16–24–32. Objective function f(x) resolution: 8 points.

**Figure A4:**
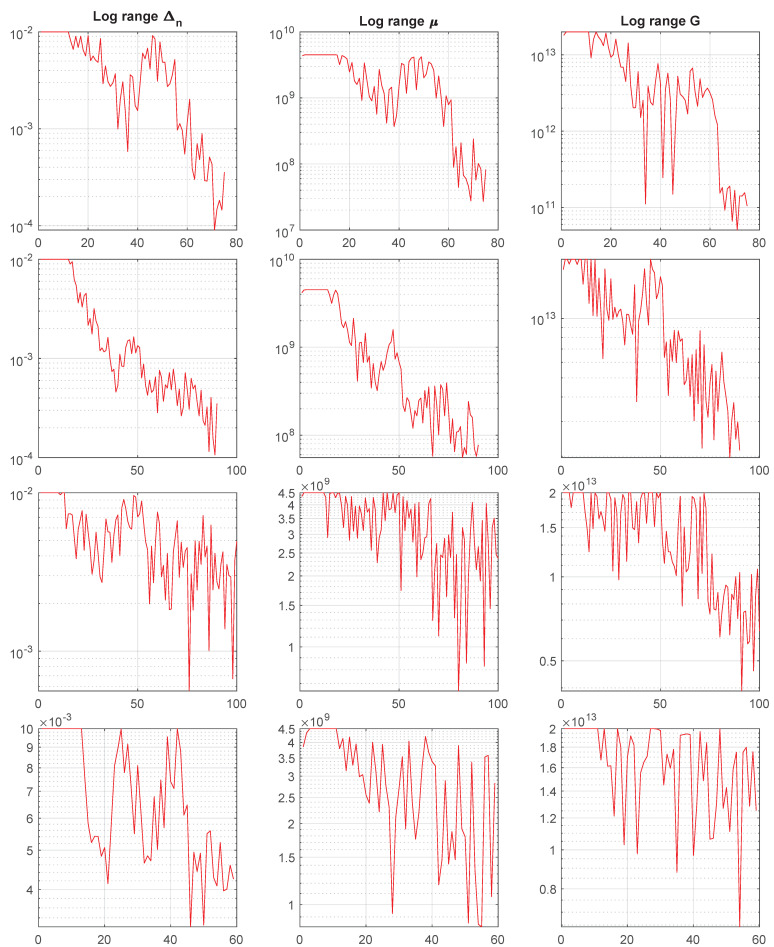
The three columns represent the evolution of each optimization variable ranges, respectively: $\Delta_{n}$, μ and *G* The horizontal axes are the PSO iterations and the vertical axes are in the units of the corresponding variable, Pa or adimentional for $\delta_{n}$. Swarm Population: 40. Objective function f(x) resolution from top to bottom: 8–16–24–32 points.

Optimizations 1, 2, 3 and 4 (Figure A1 and Figure A3) all reach the end of the optimization at iteration 100 but with poor residual value for the case 1 and 4. For the optimizations with large swarm size: 5, 6, 7 and 8 (Figure A2 and Figure A4) only the ones with objective function resolutions 16 and 32 reach convergence. Results are summarized in Table 2, last line presents the known value of *x* in bold to allow fast comparison with optimization results.

Results show some simulations, i.e., 2 and 6, converged to a very low residual but the value of *x* is still slightly off the target value; this proves that the object function presents a minimum very close to the target one around *x* = [0.0050 $9.5219\times 10^{8}$ $1.9876\times 10^{13}$]. Optimization 3 is the only one that did converge to the target value: *x* = [0.0050 $9.6105\times 10^{8}$ $2.0029\times 10^{13}$], best f(x) is not very low because the stopping criterion was reached. Next section presents a stochastic study of the micro-scale optimization.

### 4.5. Monte-Carlo Analysis

**Figure 13:**
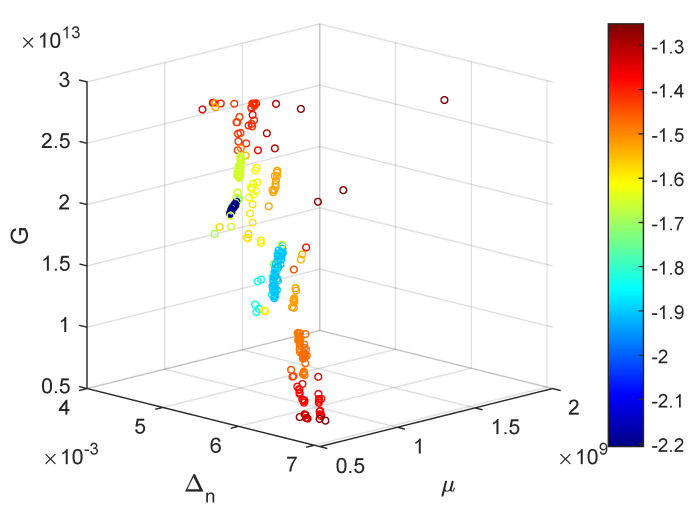
3D scatter plot in the optimization variable space, each circle is a PSO optimization, the colour represents the decimal logarithm of best *f*(*x*). The small dark blue cluster is in close proximity of the known target optimization value of *x* and contains 15 Monte-Carlo realizations.

A more in depth evaluation of the best suited values for the PSO optimization would require a complete Monte-Carlo analysis to bound the error under a desired limit; this does not fall inside the scope of this work. However, a Monte-Carlo error estimation through statistical ensemble simulations of the case #2 (Swarm Size 16 and function resolution 8) has been carried, with 273 realizations of the PSO optimization. The 3D scatter plot of the results (Figure 13) presents a banded organization of the optimizations in the $\Delta_{n}$, μ, *G* space. The results are organized in clusters which share a sensibly homogeneous value of best f(x), this observation reinforces the idea of the presence of local optima and the need of meta-heuristics.

**Figure 14:**
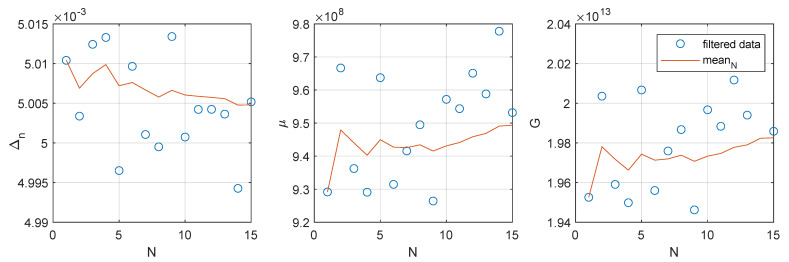
Evolution of the three optimization variable means for *N* Monte-Carlo realizations.

Figure 13 suggests that the best fit metric, f(x) can be used to discriminate between good optimizations and local optima traps. A best fit value of f(x)=0.01 has been chosen as threshold resulting in 15/273 simulations fulfilling the convergence criteria. The filtered individual and mean values $\Delta_{n}$, μ and *G* are presented (Figure 14) with *N* being the number of Monte Carlo realizations. As the sampling population *N* increases from 1 to 15 the mean value (red line) tends to the the known target values: $\Delta_{n}=0.0050$, $\mu =961\times 10^{6}$ and $G=2.00\times 10^{13}$.

**Figure 15:**
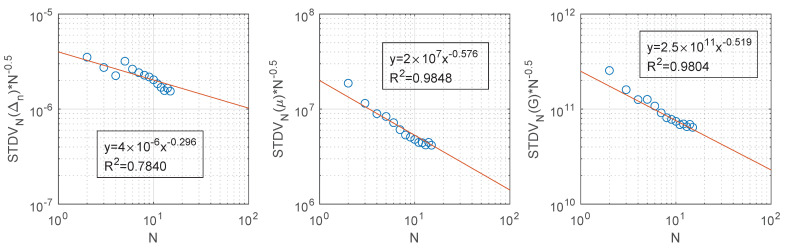
Standard deviation of the growing population *N* from 1 to 15 divided by the root square of the sampled population *N* (Blue circles). Power trend line (red line). Trend line equation and $R^{2}$ are presented with *y* representing the vertical axis $\left(STDV_{N}\left(var.\right)/N^{0.5}\right)$ and *x* the horizontal axis (N).

In order to verify convergence of the Monte-Carlo analysis, the standard deviation of the growing population *N* ($STDV_{N}$) from 1 to 15 divided by the root square of the sampled population *N* is plotted in logarithmic axes (Figure 15, blue circles), this metric can be considered as the error of a Monte-Carlo analysis. Assuming that the standard deviation of a Monte-Carlo realization is constant, the error should decrease at constant rate. Power trend lines (Figure 15, red line) present $R^{2}$ fitting values between 0.78 and 0.98, the high $R^{2}$ values confirm the convergence at constant rate. All trends present a good convergence speed with μ and *G* close to the theoretical exponent value of −0.5.

## 5. Discussion

The homogenized response of the presented micro-scale is the result of matrix-crack network interaction. For low values of crack stiffness with respect to matrix stiffness the homogenized properties are essentially determined by the first one. On the contrary, for high values of crack stiffness the homogenized coefficients are solely determined by the matrix properties. There is a range between the two extreme cases, spanning about three orders of magnitude of crack stiffness, for which the homogenized coefficients are determined by the complex interaction of both matrix and crack network. The determination of this range is important because within it, a phenomenological expression is not sufficient to obtain a valid constitutive response of the material; a double-scale model is needed.

In order to characterize the micro-structure, a series of failure surfaces have been proposed and evaluated for later optimization. Maximum force criterion has shown the presence of artifacts for the stresses applied parallel to the coordinate axes due to vanishing stress components. This has been overcome using von Misses distortion energy criterion. Due to the ultimate objective of implementing the model in a double-scale approach, yet a more suited failure envelope has been proposed which considers the sample to be failed when the modulus of the homogenized force vector loses a given ratio with respect to the peak value. This failure criterion is later used to obtain a synthetic failure envelope that serves as an optimization target.

Typical elastoplastic constitutive laws present continuous and differentiable yield surfaces, e.g., Druker-Praguer. The differentiability assumption allows to calibrate them with biaxial/triaxial test results using simple fitting techniques and ultimately gradient methods. Previous assumptions are not applicable to constitutive laws such as the one used in this work. Meta-heuristics have been proposed; PSO is used to minimize the objective function consisting in the fitting of the micro-scale numerical results to the synthetic failure envelope. PSO is not immune to local optima traps and indeed it converged to some of those local minima far from the actual solution in some of the optimizations, other runs converged to a value very close to the target one due to the presence of a local minimum in the vicinity of the target. Among the 8 optimizations at least one converged to the target values within 100 iterations of PSO. Since PSO is a probabilistic method, the lower accuracy of small swarm sizes can be compensated by running a higher number of optimizations (in a Monte-Carlo fashion), this allows the user to decide when to stop the process thus saving time. Higher swarm sizes can be used when computational load or time are less restrictive and accuracy is preferred over speed. The range of function resolutions used (8–64 points) shows the ability of the approach to optimize a constitutive law with different available material data. The metric f(x) has successfully been used to discriminate between local and global optima in a complete Monte-Carlo analysis for the case with swarm size 16 and resolution 8. With an error defined by $STDV_{N}/\sqrt{N}$, filtered results of the Monte-Carlo analysis confirm convergence towards the global optimum.

Results prove that: given a target failure curve, PSO is able to optimize the parameters of a constitutive law issued from an elastic matrix with damageable crack network. And in a more general sense: meta-heuristics can be used to optimize complex constitutive laws in multi-scale numerical schemes. Future works should focus on the improvement of PSO settings to accelerate convergence and avoidance of local minima (robustness), use of real response curves instead of synthetic ones, use of other meta-heuristics and Machine Learning Algorithms.

## 6. Conclusions

This work presented a micro-scale model for multi-scale use, established its validity range based on *in-materio* computing and provided a meta-heuristics calibration method. Specific conclusions are as follows:

- A ratio to peak stress has shown to be a good criteria to characterize the failure of the present micro-structure.
- For the initially given λ and μ elastic coefficients, the multi-scale rich micro-structure behaviour happens in the crack stiffness range $G=[1.0\times 10^{13}$∼$2.7\times 10^{13}]$ Pa.
- PSO overcomes the non continuity and non differentiability of the constitutive law for a representative range of function resolutions.
- The metric best f(x) alone is able to discriminate between local and global optima.

The presented micro-structure can be applied to model a range of blocky or fractured media such as coal, crystalline rocks and composites with applications ranging from coal mining, CO${}_{2}$ sequestration, coalbed methane extraction, deep geological nuclear waste disposal, to masonry structures.

### Contribution to Science

Multi-scale numerical methods have experienced a revived interest in the last decades thanks to the increase of available computing power. The present paper provides an insight to the properties and limitations of multi-scale targeted micro-scale models. This can help researchers to determine whether or not a micro-scale description is needed in a particular model. Furthermore the meta-heuristics optimization paves the way to further investigate the use of AI paradigms to better characterize the micro-scale of materials.

## Figures and Tables

**Table 1:** Particle Swarm Optimization parameters.

PSO Parameter	Value
Function Tolerance	$1.0\times 10^{-6}$
Inertia Range	[0.1000 1.1000]
Min. Neighbors Fraction	0.25
Objective Limit	0.0
Self Adjustment Weight	1.4900
Social Adjustment Weight	1.4900

**Table 2:** Particle Swarm Optimization simulation summary and results. Run in a computer with Intel Core i7-10700 processor @2.90 GHz and 16.0 GB RAM @2933 MHz, all simulations using parallelization with 8 workers.

Sim.	Swarm Size	It.	f-Count	*f*(*x*) Res.	Best *f*(*x*)	Mean *f*(*x*)	Optimal *x*	Time
1	8	100	808	8	0.01245	0.03517	[0.0056 9.4330 $\times 10^{8}$ 1.6802 $\times 10^{13}$]	42 min
2	16	100	1616	8	$2.608\times 10^{-6}$	0.003639	[0.0050 9.5287 $\times 10^{8}$ 1.9847 $\times 10^{13}$]	90 min
3	24	100	2424	8	0.0002951	0.06567	[0.0050 9.6105 $\times 10^{8}$ 2.0029 $\times 10^{13}$]	135 min
4	32	100	3232	8	0.0379	0.1722	[0.0064 6.6052 $\times 10^{8}$ 1.0255 $\times 10^{13}$]	170 min
5	40	75	3040	8	0.02985	0.03623	[0.0044 1.1140 $\times 10^{9}$ 2.6967 $\times 10^{13}$]	3 h
6	40	100	3640	16	$6.136\times 10^{-10}$	0.00424	[0.0050 9.5219 $\times 10^{8}$ 1.9876 $\times 10^{13}$]	6 h
7	40	100	4040	32	$1.635\times 10^{-8}$	0.1146	[0.0050 9.6970 $\times 10^{8}$ 2.0132 $\times 10^{13}$]	14 h
8	40	59	2400	64	0.09868	0.1690	[0.0049 1.2485 $\times 10^{9}$ 2.5521 $\times 10^{13}$]	17 h
**Ref. x**	-	-	-	-	-	-	**[0.0050 9.6100 $\times 10^{8}$ 2.0000 $\times 10^{13}$]**	-

## Data Availability

The data presented in this study are available on request from the corresponding author.

## Supplementary Materials

The following are available online at https://doi.org/10.6084/m9.figshare.14540118.v1 (posted on 5 May 2021).

## Abbreviations

The following abbreviations are used in this manuscript:

PDE	Partial Differential Equation
AI	Artificial Intelligence
ML	Machine Learning
FEM	Finite Element Model
PSO	Particle Swarm Optimization

## References

[1] Auriault J.L. (1991). Heterogeneous medium. Is an equivalent macroscopic description possible?. Int. J. Eng. Sci..

[2] Biot M. (1941). General theory of three-dimensional consolidation. J. Appl. Phys..

[3] Chambon R., Caillerie D., Viggiani G. (2004). Loss of uniqueness and bifurcation vs instability: Some remarks. Rev. Fr. Génie Civ..

[4] Papanicolau G., Bensoussan A., Lions J.L. (1978). Asymptotic Analysis for Periodic Structures.

[5] Sánchez-Palencia E. (1980). Non-Homogeneous Media and Vibration Theory.

[6] Arbogast T., Douglas J., Hornung U. (1990). Derivation of the double porosity model of single phase flow via homogenization theory. SIAM J. Math. Anal..

[7] Bensoussan A., Lions J.L., Papanicolaou G. (2011). Asymptotic Analysis for Periodic Structures.

[8] Sridhar A., Kouznetsova V., Geers M. (2017). A general multiscale framework for the emergent effective elastodynamics of metamaterials. J. Mech. Phys. Solids.

[9] Waseem A., Heuzé T., Stainier L., Geers M., Kouznetsova V. (2020). Model reduction in computational homogenization for transient heat conduction. Comput. Mech..

[10] Auriault J.L. (2011). Heterogeneous periodic and random media. Are the equivalent macroscopic descriptions similar?. Int. J. Eng. Sci..

[11] Stefanou I., Sulem J., Vardoulakis I. (2008). Three-dimensional Cosserat homogenization of masonry structures: Elasticity. Acta Geotech..

[12] Godio M., Stefanou I., Sab K., Sulem J., Sakji S. (2017). A limit analysis approach based on Cosserat continuum for the evaluation of the in-plane strength of discrete media: Application to masonry. Eur. J. Mech. A Solids.

[13] Scholtès L., Donzé F.V., Khanal M. (2011). Scale effects on strength of geomaterials, case study: Coal. J. Mech. Phys. Solids.

[14] Bertrand F., Cerfontaine B., Collin F. (2017). A fully coupled hydro-mechanical model for the modeling of coalbed methane recovery. J. Nat. Gas Sci. Eng..

[15] Valle V., Hedan S., Cosenza P., Fauchille A.L., Berdjane M. (2015). Digital image correlation development for the study of materials including multiple crossing cracks. Exp. Mech..

[16] Ougier-Simonin A., Renard F., Boehm C., Vidal-Gilbert S. (2016). Microfracturing and microporosity in shales. Earth-Sci. Rev..

[17] Arson C. (2020). Micro-macro mechanics of damage and healing in rocks. Open Geomech..

[18] Dale M., Miller J., Stepney S. (2017). Reservoir Computing as a Model for In-Materio Computing.

[19] Germain P. (1973). La méthode des puissances virtuelles en mécanique des milieux continus. J. Mec..

[20] Papachristos E., Stefanou I. (2021). Controlling earthquake-like instabilities using artificial intelligence. arXiv.

[21] Masi F., Stefanou I., Vannucci P., Maffi-Berthier V. (2021). Thermodynamics-based Artificial Neural Networks for constitutive modeling. J. Mech. Phys. Solids.

[22] Lu X., Giovanis D.G., Yvonnet J., Papadopoulos V., Detrez F., Bai J. (2019). A data-driven computational homogenization method based on neural networks for the nonlinear anisotropic electrical response of graphene/polymer nanocomposites. Comput. Mech..

[23] Wang K., Sun W. (2018). A multiscale multi-permeability poroplasticity model linked by recursive homogenizations and deep learning. Comput. Methods Appl. Mech. Eng..

[24] Logarzo H.J., Capuano G., Rimoli J.J. (2021). Smart constitutive laws: Inelastic homogenization through machine learning. Comput. Methods Appl. Mech. Eng..

[25] Liu Z., Wu C. (2019). Exploring the 3D architectures of deep material network in data-driven multiscale mechanics. J. Mech. Phys. Solids.

[26] Jang D.P., Fazily P., Yoon J.W. (2021). Machine learning-based constitutive model for J2-plasticity. Int. J. Plast..

[27] Vlassis N.N., Sun W. (2021). Sobolev training of thermodynamic-informed neural networks for interpretable elasto-plasticity models with level set hardening. Comput. Methods Appl. Mech. Eng..

[28] Raissi M., Perdikaris P., Karniadakis G.E. (2019). Physics-informed neural networks: A deep learning framework for solving forward and inverse problems involving nonlinear partial differential equations. J. Comput. Phys..

[29] Raissi M., Yazdani A., Karniadakis G. (2020). Hidden fluid mechanics: Learning velocity and pressure fields from flow visualizations. Science.

[30] Ye H., Shen Z., Xian W., Zhang T., Tang S., Li Y. (2020). OpenFSI: A highly efficient and portable fluid–structure simulation package based on immersed-boundary method. Comput. Phys. Commun..

[31] Jokar M., Semperlotti F. (2021). Finite element network analysis: A machine learning based computational framework for the simulation of physical systems. Comput. Struct..

[32] Miller J., Harding S., Tufte G. (2014). Evolution-in-materio: Evolving computation in materials. Evol. Intell..

[33] Beggs J. (2008). The criticality hypothesis: How local cortical networks might optimize information processing. Philos. Trans. Ser. A Math. Phys. Eng. Sci..

[34] Gibbons T. (2010). Unifying Quality Metrics for Reservoir Networks.

[35] Meier H., Steinmann P., Kuhl E. (2008). Towards multiscale computation of confined granular media. Tech. Mech..

[36] de Souza Neto E., Blanco P., Sánchez P., Feijóo R. (2015). An RVE-based multiscale theory of solids with micro-scale inertia and body force effects. Mech. Mater..

[37] Liu Y., Sun W., Yuan Z., Fish J. (2015). A nonlocal multiscale discrete-continuum model for predicting mechanical behavior of granular materials. Int. J. Numer. Methods Eng..

[38] van den Eijnden B., Bésuelle P., Chambon R., Collin F. (2016). A FE2 modelling approach to hydromechanical coupling in cracking-induced localization problems. Int. J. Solids Struct..

[39] Desrues J., Argilaga A., Caillerie D., Combe G., Nguyen T., Richefeu V., Dal Pont S. (2019). From discrete to continuum modelling of boundary value problems in geomechanics: An integrated FEM-DEM approach. Int. J. Numer. Anal. Methods Geomech..

[40] Guo N., Yang Z., Yuan W.H., Zhao J. (2020). A coupled SPFEM/DEM approach for multiscale modeling of large-deformation geomechanical problems. Int. J. Numer. Anal. Methods Geomech..

[41] Argilaga A., Papachristos E., Caillerie D., Dal Pont S. (2016). Homogenization of a cracked saturated porous medium: Theoretical aspects and numerical implementation. Int. J. Solids Struct..

[42] Pardoen B., Bésuelle P., Dal Pont S., Cosenza P., Desrues J. (2020). Accounting for Small-Scale Heterogeneity and Variability of Clay Rock in Homogenised Numerical Micromechanical Response and Microcracking. Rock Mech. Rock Eng..

[43] Viggiani G., Andò E., Takano D., Santamarina J. (2015). Laboratory X-ray Tomography: A Valuable Experimental Tool for Revealing Processes in Soils. Geotech. Test. J..

[44] Lukić B., Tengattini A., Dufour F., Briffaut M. (2021). Visualising water vapour condensation in cracked concrete with dynamic neutron radiography. Mater. Lett..

[45] Majmudar T., Behringer R. (2005). Contact force measurements and stress-induced anisotropy in granular materials. Nature.

[46] Wiebicke M., Andò E., Herle I., Viggiani G. (2017). On the metrology of interparticle contacts in sand from X-ray tomography images. Meas. Sci. Technol..

[47] Wiebicke M., Ando E., Šmilauer V., Herle I., Viggiani G. (2019). A benchmark strategy for the experimental measurement of contact fabric. Granul. Matter.

[48] Argilaga A., Desrues J., Dal Pont S., Combe G., Caillerie D. (2018). FEMxDEM multiscale modeling: Model performance enhancement, from Newton strategy to element loop parallelization. Int. J. Numer. Methods Eng..

[49] Mirjalili S.Z., Mirjalili S., Saremi S., Faris H., Aljarah I. (2018). Grasshopper optimization algorithm for multi-objective optimization problems. Appl. Intell..

[50] Heidari A.A., Faris H., Aljarah I., Mirjalili S., Mafarja M., Chen H. (2019). Harris hawks optimization: Algorithm and applications. Future Gener. Comput. Syst..

[51] Li S., Chen H., Wang M., Heidari A.A., Mirjalili S. (2020). Slime mould algorithm: A new method for stochastic optimization. Future Gener. Comput. Syst..

[52] Kennedy J., Sammut C., Webb G.I. (2010). Particle Swarm Optimization. Encyclopedia of Machine Learning.

[53] Royer P., Cherblanc F. (2010). Homogenisation of advective–diffusive transport in poroelastic media. Mech. Res. Commun..

[54] Auriault J.L. (2005). Transport in porous media: Upscaling by multiscale asymptotic expansions. Applied Micromechanics of Porous Materials.

[55] Dascalu C., Bilbie G., Agiasofitou E. (2008). Damage and size effects in elastic solids: A homogenization approach. Int. J. Solids Struct..

[56] Marinelli F., Sieffert Y., Chambon R. (2015). Hydromechanical modeling of an initial boundary value problem: Studies of non-uniqueness with a second gradient continuum. Int. J. Solids Struct..

[57] Hill R. (1962). Acceleration waves in solids. J. Mech. Phys. Solids.

[58] Rice J.R. (1976). The Localization of Plastic Deformation.

[59] Mindlin R.D. (1965). Second gradient of strain and surface-tension in linear elasticity. Int. J. Solids Struct..

[60] Chambon R., Caillerie D., El Hassan N. (1998). One-dimensional localisation studied with a second grade model. Eur. J. Mech. A/Solids.

[61] Chambon R., Caillerie D., Matsuchima T. (2001). Plastic continuum with microstructure, local second gradient theories for geomaterials: Localization studies. Int. J. Solids Struct..

[62] Collin F., Chambon R., Charlier R. (2006). A finite element method for poro mechanical modelling of geotechnical problems using local second gradient models. Int. J. Numer. Methods Eng..

[63] Nova R. (1994). Controllability of the incremental response of soil specimens subjected to arbitrary loading programmes. J. Mech. Behav. Mater..

